# Accuracy of advanced deep learning with tensorflow and keras for classifying teeth developmental stages in digital panoramic imaging

**DOI:** 10.1186/s12880-022-00794-6

**Published:** 2022-04-08

**Authors:** Norhasmira Mohammad, Anuar Mikdad Muad, Rohana Ahmad, Mohd Yusmiaidil Putera Mohd Yusof

**Affiliations:** 1 Center for Oral and Maxillofacial Diagnostics and Medicine Studies, Faculty of Dentistry, Universiti Teknologi MARA, Sungai Buloh Campus, 47000 Sungai Buloh, Selangor Malaysia; 2 Center for Integrated Systems Engineering and Advanced Technologies (INTEGRA), Faculty of Engineering and Built Environment, Universiti Kebangsaan Malaysia (UKM), 43600 Bangi, Selangor Malaysia; 3 Center for Restorative Dentistry Studies, Faculty of Dentistry, Universiti Teknologi MARA, Sungai Buloh Campus, 47000 Sungai Buloh, Selangor Malaysia; 4 Institute of Pathology, Laboratory and Forensic Medicine (I-PPerForM), Universiti Teknologi MARA, Sungai Buloh Campus, 47000 Sungai Buloh, Selangor Malaysia

**Keywords:** Deep learning, Tooth segmentation, Premolar, Digital dental panoramic radiographs

## Abstract

**Background:**

This study aims to propose the combinations of image processing and machine learning model to segment the maturity development of the mandibular premolars using a Keras-based deep learning convolutional neural networks (DCNN) model.

**Methods:**

A dataset consisting of 240 images (20 images per stage per sex) of retrospect digital dental panoramic imaging of patients between 5 and 14 years of age was retrieved. In image preprocessing, abounding box with a dimension of 250 × 250 pixels was assigned to the left mandibular first (P1) and second (P2) permanent premolars. The implementation of dynamic programming of active contour (DP-AC) and convolutions neural network on images that require the procedure of image filtration using Python TensorFlow and Keras libraries were performed in image segmentation and classification, respectively.

**Results:**

Image segmentation using the DP-AC algorithm enhanced the visibility of the image features in the region of interest while suppressing the image's background noise. The proposed model has an accuracy of 97.74%, 96.63% and 78.13% on the training, validation, and testing set, respectively. In addition, moderate agreement (Kappa value = 0.58) between human observer and computer were identified. Nonetheless, a robust DCNN model was achieved as there is no sign of the model's over-or under-fitting upon the learning process.

**Conclusions:**

The application of digital imaging and deep learning techniques used by the DP-AC and convolutions neural network algorithms to segment and identify premolars provides promising results for semi-automated forensic dental staging in the future.

## Background

Age estimation is essential in forensic medicine for identifying deceased victims and assessing the age of those involved in crimes and accidents. Forensic odontology is one of the forensic branches used for human identification through teeth. It is a term that refers to a variety of clinical, analytical, radiographic, and other methods for estimating dental age, which is then converted to chronological age. Since 1982, dental radiographic imaging, as obtained by the dental x-ray equipment, has been used in age estimation approaches as a non-destructive and straightforward technology used in dental practice [1]. In addition, the age estimation technique based on dental development has been widely used in other clinical set-up like the orthodontics and paediatric dentistry. This method entails identifying the mineralization stage on radiographic images and comparing it to a standard stage to determine the approximate age range [2, 3].

Demirjian’s method is the most commonly used method for age estimation and has gained worldwide acceptability due to its objective criteria for describing the stages of tooth development [4–6]. This method involves human expertise to assess eight stages of dental development of the seven left permanent mandibular teeth. Then, each tooth stage is assigned biologic weights, which are numerical and computed following the method described in study on skeletal maturity [7]. Next, the dental maturity score is calculated by combining the weights. Finally, to convert maturity scores to dental age, separate tables of dental maturity for males and girls [4] are employed. The conventional way of estimating an individual's age can be an intricate procedure involving a significant number of forensic identifications, especially in the pandemic situation. Hence, automation of the conventional method may serve as a starting point for improving efficiency and reproducibility during identification process.

Digital image processing and deep learning techniques are applied to resolve the matters concerning the conventional method of forensic dental age estimation especially during large scale calamities for disaster victim identification. Issues such as the time taken to estimate age using manual atlas, subjective scoring due to operator’s bias, and large number of cases to be completed within short period are among the reasons to expedite the use of semi-automated or fully automated dental scoring. Recent publications related to the segmentation of x-ray images for multipurpose medical application have been reported. Segmentation categories can be divided into four which are region-based, cluster-based, threshold-based and watershed-based segmentation. For region-based segmentation approach, dynamic programming of gradient inverse coefficient of variation (DP-GICOV) and Chan-Vese (CV) was employed in Muad et al. to perform a segmentation of mandibular third molar tooth for forensic human identification [8]. Results demonstrated that DP-GICOV performs a better segmentation with an accuracy of 95.3%. A method based on clustering segmentation has been presented where a novel semi-supervised fuzzy clustering algorithm with spatial constraints for dental segmentation is proposed [9]. The result shows that the proposed method performs a better segmentation result compared to semi-supervised fuzzy clustering and other relevant methods. Hence, a new parameter has been suggested by the author for better performance.

For the threshold-based method, a novel semi-supervised Hyperbolic Tangent Gaussian kernel fuzzy C-Means clustering (HTGkFCM)-Otsu method is proposed in Kumar et al. [10]. The hyperbolic tangent Gaussian kernel method associates the input data space nonlinearly into attribute space having high dimensional and less sensitive to noise with robustness while adding kernel information to the proposed algorithm enhanced the original FCM algorithm. Watershed-based segmentation method has been widely tested on countless medical images and produced promising results. Recent studies on this method have been presented in various publications [11–13]. This approach's fundamental is based on the morphological mathematics operation where the visualization of a grey level in the images is transformed into a topographic representation that includes three basic concepts: local minima, catchment basins and watershed lines. The computation of marker image plays a significant role in determining a good segmentation output and minimalizing the over-segmentation result.

The deep learning convolutional neural networks (DCNN) have recently been introduced to perform automated tooth segmentation where these artificial intelligence methods have shown better performance than other mathematical approaches. A pilot study to stage lower third molar development on panoramic radiographs for age estimation was proposed in De Tobel et al. where the pre-trained AlexNet network model was adapted [14]. However, improvements have been made using DenseNet201 network for automated stage allocation [15]. Based on the improvement, the authors made a new hypothesis that only the third molar could improve the automated stage allocation performance. As of now, there are few studies utilizing DCNN in their methods [16, 17]. In the dental age estimation, literature stated that the third molar variation might affect age estimation accuracy on different populations [18–20]. The automated approach, the classification accuracy may also be affected due to the tooth's morphological and its surrounding. The unwanted object, such as periodontal ligament, bony structures, and mandibular nerve canal, influences automated stage allocation performance, as stated by Boedi et al. [15]. Following several systematic reviews on dental age estimation methods among children and adolescents, the use of premolar development stages as one of the variables accurately estimate the age with less than one year based on the margin of error [21, 22]. These studies, however, were based on conventional radiographic method of dental age estimation.

**Fig. 1:**
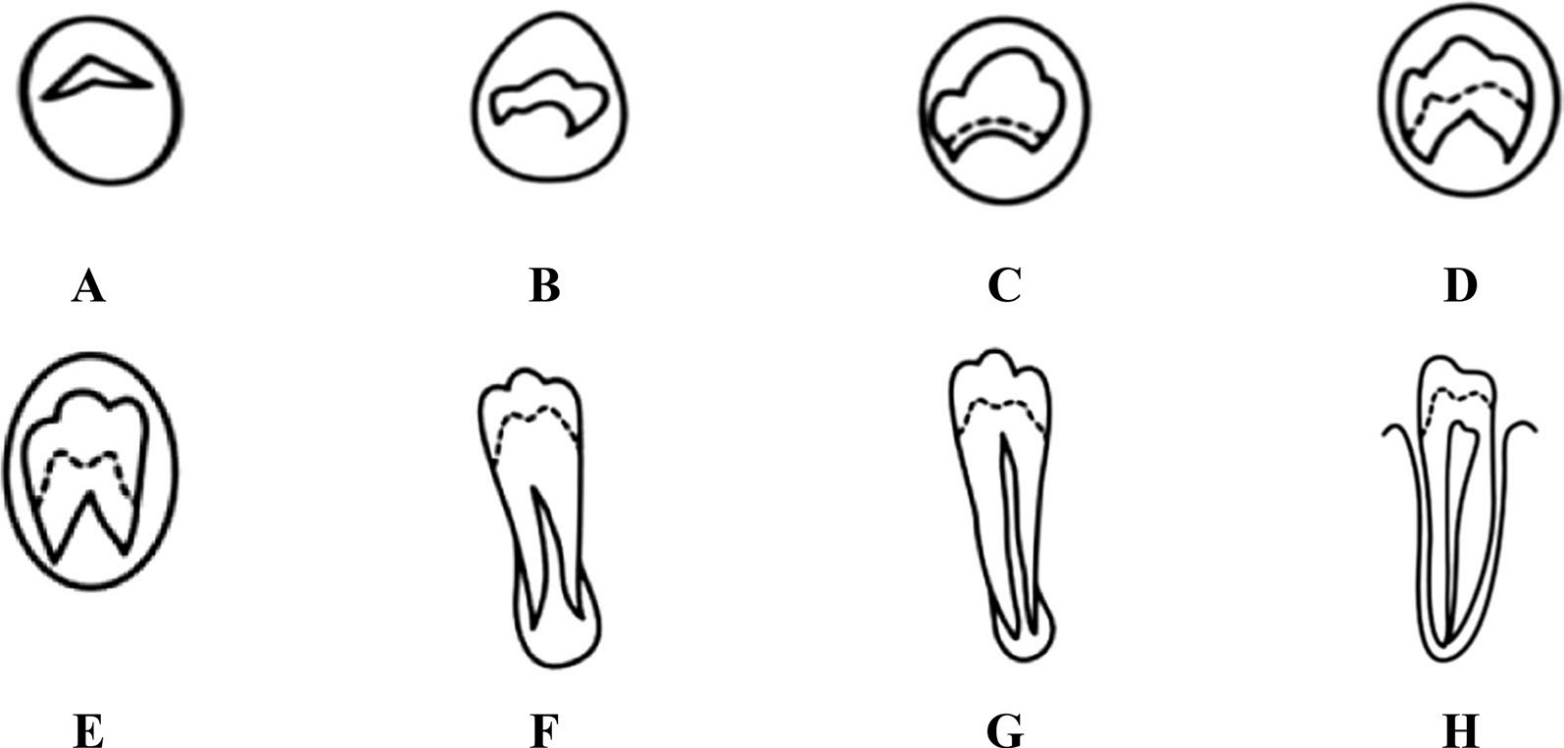
Demirjian stages of tooth development [4, 24] Stage **A**: uncoordinated mineralized cusp tips; stage **B**: united mineralized cusps; stage **C**: crown formation is about halfway complete; stage **D**: crown formation is complete to the dento-enamel junction; stage **E**: root formation has begun; stage **F**: root length equals crown length; stage **G**: parallel root walls with open apices; stage **H**: apices are completely closed

Therefore, the present pilot study aimed to observe the feasibility of both combinations of image processing and machine learning approach to segment and classify the maturity development of the mandibular premolars using a DCNN model. As these monoradicular teeth have less variation than the third molar, the pilot segmentation of premolars tooth may improve the proposed method's performance. The current study is an extension from the previous study by Mohammad et al. to enhance the existing segmentation and classification method [23]. In addition, Demirjian’s staging system is adopted in which premolar teeth will be segmented and classified accordingly following the atlas, as shown in Fig. 1.

## Proposed methodology

**Fig. 2:**
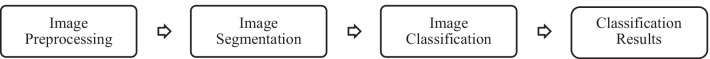
Proposed framework

In this study, a new approach based on digital image processing and DCNN technique is applied to solve the original work issues. A dataset which consists of twenty radiographs per stage per sex were retrospectively selected. The methodologies include three phases which are image preprocessing, image segmentation and image classification. Figure 2 shows the sequence operation of the methodology. This study protocols have been reviewed and approved by the local institutional review board. The patients’ informed consent was waived.

### Image preprocessing

Image preprocessing includes the extraction of the region of interest (ROI) and image enhancement technique. Abounding box with a dimension of 250 × 250 pixels is assigned to all the mandibular first (P1) and second (P2) permanent premolars in panoramic dental radiographs where the object is approximately located at the centre of the image. Then, the intensity of the image is adjusted, and the median filter is applied. This operation works by highlighting the foreground and suppressing the image's background, which will help later processing. A median filter is used to remove an impulsive noise while preserving the object edges where a kernel filter with a size of 7 × 7 is applied.

### Image segmentation

Active contour (AC) is one of the active models in segmentation techniques, which uses the image's energy constraints and forces to separate the region of interest. AC outlines a distinct boundary or curvature in the target region for segmentation. The basic computation of AC is based on optimizing a cost function where two main approaches were developed from the calculus of variations and dynamic programming (DP) [25]. The approach has the capability of dealing with a variety of cost functions. However, it depends on gradient descent methods for local optimization and requires a good initialization for the contour whilst DP-AC computation does not require contour initialization, but it needs a confined form of the cost function [26].

**Fig. 3:**
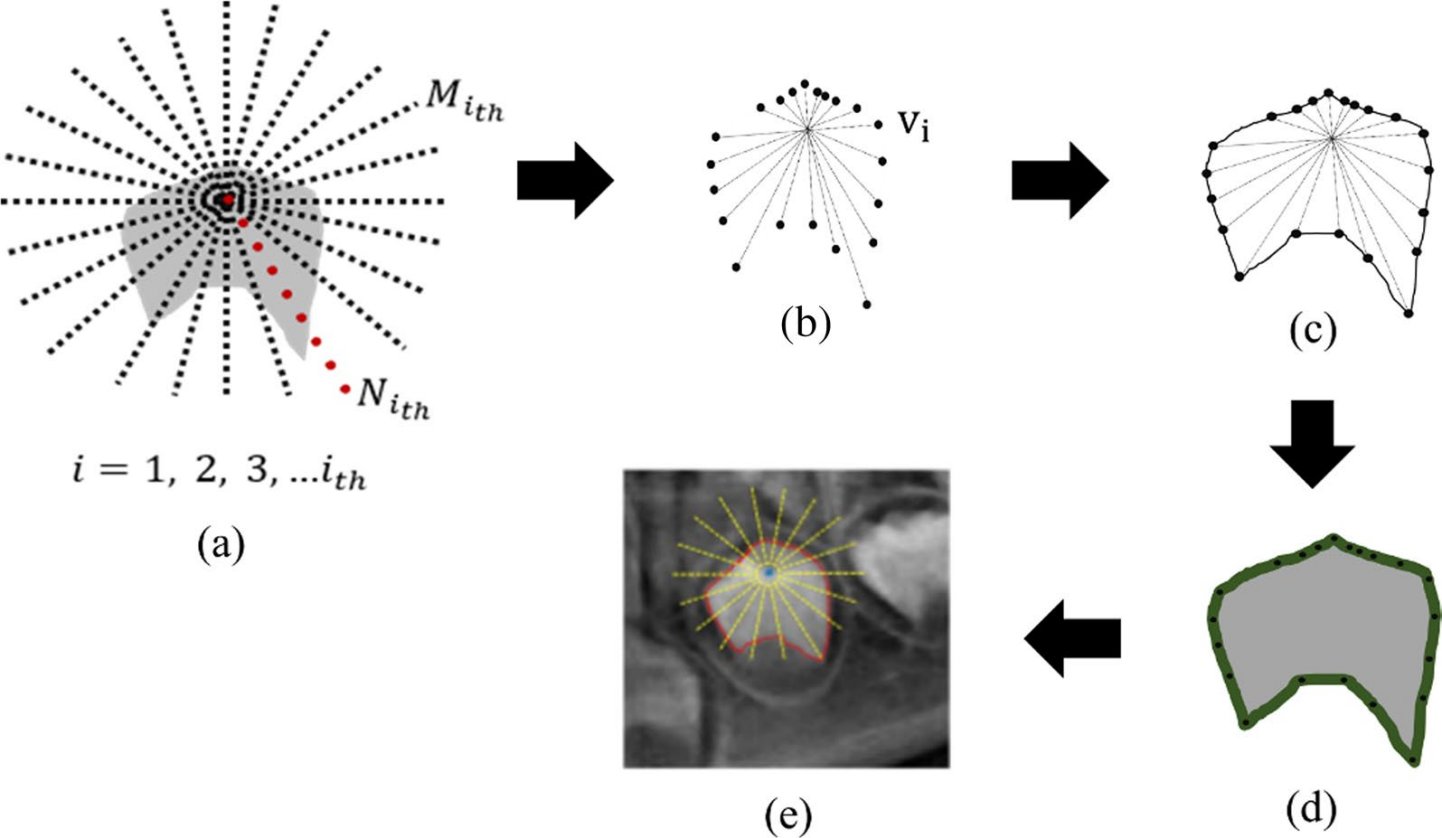
The example of constructed radial lines in the schematic diagram forms a closed contour on ROI. Image **a** depicts the assumption of radial lines emerging from the centroid, **b** the detected $N{i}_{th}$ point on ${M}_{{i}_{th}}$, **c** the contour with the greatest directional gradient strength, (d) the region mask obtained after performing polygon to binary conversion, and **e** delineated ROI based on a region mask on the original input image

For object delineation and segmentation, the DP-AC implementation is based on the formula proposed in the study by Ray et al. [27]. The original work was done to perform the particle object segmentation, a leukocyte in a microscopic image. To begin the segmentation process, as the ROI is located central to the segmentation setting, concerning Fig. 3e, an assumption is made by assuming a point inside the object and emerging radial lines come out from that point. Each radial line will intersect the object boundary only once. Based on Fig. 3a, assume there are $M$ radial lines and on each $M$, let there be $N$ discrete points. Therefore, a constructed $MN$ different closed contours are obtained, in which programming uses only one point in each of $M$ lines which denotes as ${v}_{i}$, shown in Fig. 3b. By ${v}_{i}$ we denote a variable, which can take any of the $N$ discrete graduations on the ${i}_{th}$ radial line. The following equality is a typical overlapping and additive form of a cost function that DP can optimize in $O\left( {NM^{2} } \right)$ computations:

1 $$E\left( {v_{1} , v_{2} , . . . , v_{N} } \right) = E_{1} \left( {v_{1} , v_{2} } \right) + E_{2} \left( {v_{2} , v_{3} } \right) + \cdots E_{N - 1} \left( {v_{N - 1} , v_{N} } \right) + E_{N} \left( {v_{N} ,v_{1} } \right)$$

If $g\left( {v_{i} } \right)$ denotes the directional image gradient computed at location $v_{i}$, each additive cost component $E_{i}$ can be defined as:

2 $$E_{i} \left( {v_{i} ,v_{i + 1} } \right) = \left\{ {\begin{array}{*{20}l} { - g\left( {v_{i} } \right)} \hfill & {if\;D\left( {v_{i} ,v_{i + 1} } \right) \le \delta } \hfill \\ \infty \hfill & {Otherwise } \hfill \\ \end{array} } \right.$$

**Fig. 4:**
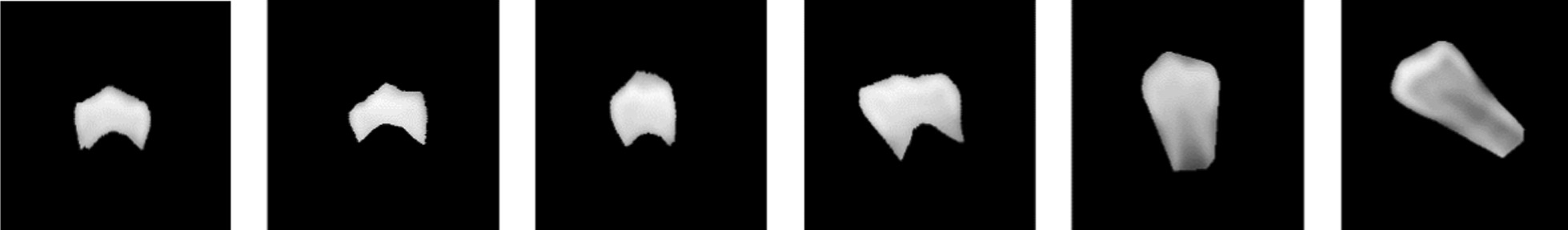
Results of superimpose the DP-AC output to the original image

The cost component (2) implies that if the distance $D$ between two consecutive points on the AC are within a certain user-defined distance $\delta$, then the cost is the negative of the directional gradient; otherwise, the cost is assigned a large value. With (2) as the individual cost component, the net effect of minimizing (1) would be to obtain a contour with the maximum directional gradient strength and with any two consecutive contour points within $\delta$ distance. The end effect is a smooth contour as shown in Fig. 3c. Next, the ROI is extracted by converting polygon to a region mask in which the output shown in Fig. 3d. The mask image is then superimposed with the original image to obtain only the ROI without background objects. The image was saved in the JPEG file for later processing. Figure 4 depicts some random samples of results obtained after the superimposition procedure. These images will undergo the learning process using the classification algorithm, which is discussed in the next section.

### Image classification

**Fig. 5:**
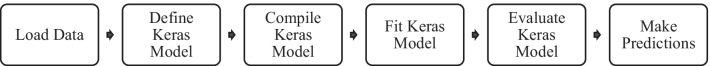
Implementation of DCNN for dental stage classification

Multi-class classification with the Python Deep Learning Library is implemented in order to perform image classification. Figure 5 demonstrates the steps taken to implement DCNN using the Python TensorFlow and Keras libraries.

**Fig. 6:**
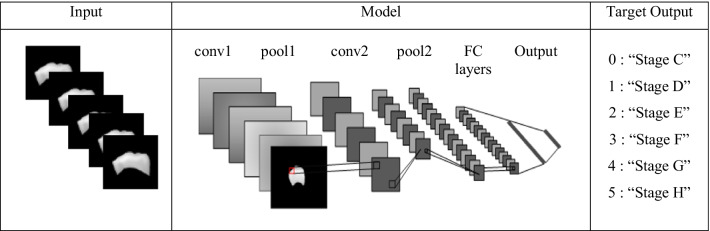
Basic Structure of DCNN

Figure 6 shows the graphical representation of the DCNN structure, where it consists of convolution (conv), pooling (pool) and fully connected (FC) layers. The input images consist of the segmented image of P1 and P2 are fed into the DCNN model where the classification takes place at the FC layer with an arbitrary output of 0 to 5, which involves six stages of dental growth (stage C to stage H). Eighty per cent of the dataset was allocated for training and validation, while the remaining 20 per cent was allocated for testing. As the available dataset consists of only 240 images, the data augmentation techniques are applied before the image classification is carried out. A new dataset consisting of 2400 images (200 images per stage per sex) is therefore obtained. The DCNN model with 3-convolutional layers-64 nodes-2 dense layers were implemented in this report. Table 1 indicates the parameter assigned to DCNN architecture. Other parameters set for the experiment; optimizer = "Adam," epoch number = 10 and batch size = 8.

**Table 1:** Defining DCNN model parameter

Layer (type)	Output shape	Parameter
conv2d (Conv2D)	(None, 90, 90, 64)	640
activation (Activation)	(None, 90, 90, 64)	0
conv2d_1 (Conv2D)	(None, 30, 30, 64)	36,928
activation_1 (Activation)	(None, 30, 30, 64)	0
max_pooling2d (MaxPooling2D)	(None, 15, 15, 64)	0
conv2d_2 (Conv2D)	(None, 15, 15, 64)	36,928
activation_2 (Activation)	(None, 15, 15, 64)	0
conv2d_3 (Conv2D)	(None, 5, 5, 64)	36,928
activation_3 (Activation)	(None, 5, 5, 64)	0
max_pooling2d_1 (MaxPooling2	(None, 2, 2, 64)	0
flatten (Flatten)	(None, 256)	0
dense (Dense)	(None, 30)	7710
activation_4 (Activation)	(None, 30)	0
dense_1 (Dense)	(None, 6)	186
activation_5 (Activation)	(None, 6)	0

Total parameters: 119,320

Trainable parameters: 119,320

Non-trainable parameters: 0

## Results

### Premolars segmentation

**Fig. 7:**
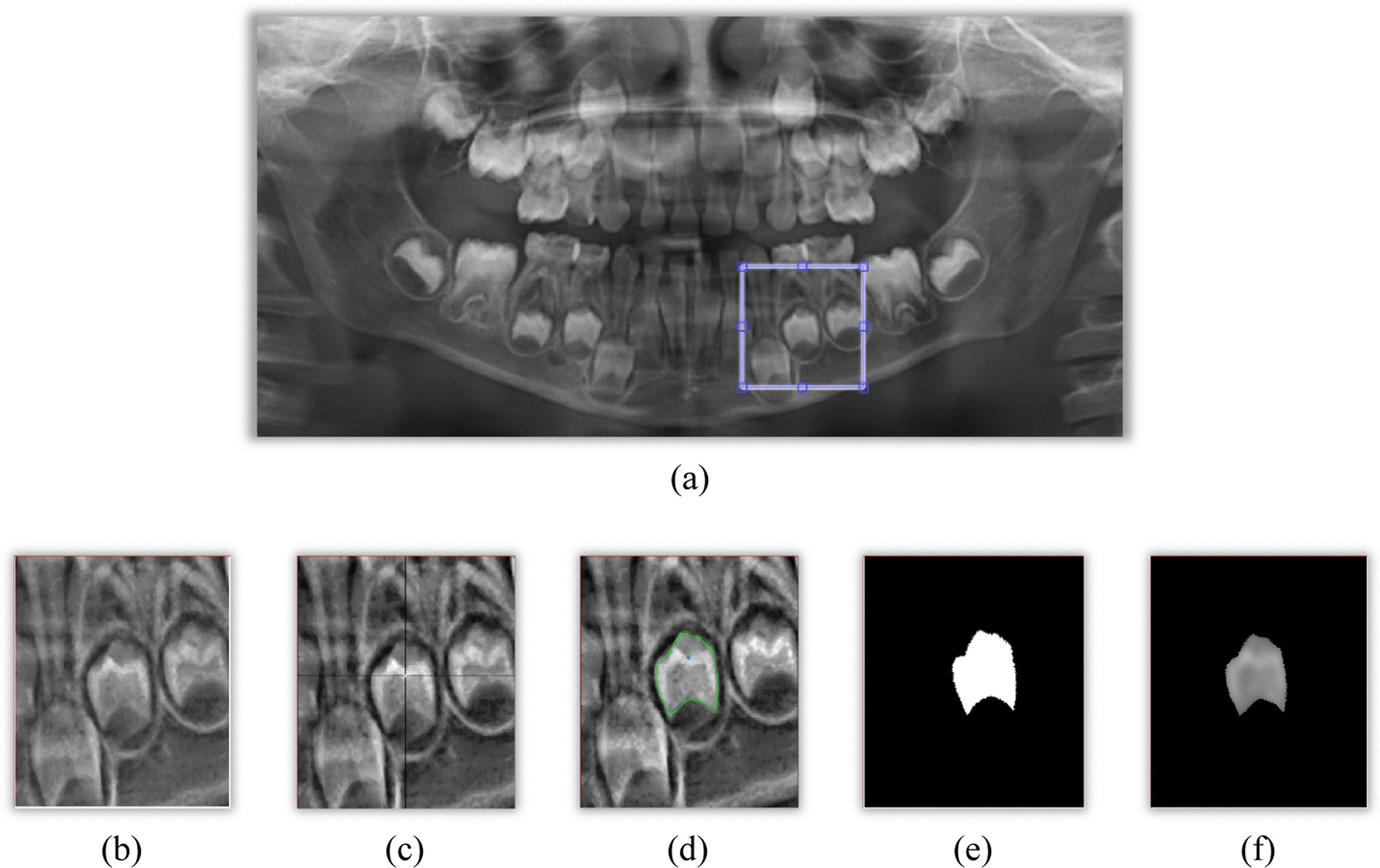
Output images based on the sequence steps of segmentation operation. Image **a** shows the placement of boundary box on the region of interest, **b** obtained after applying the crop function and enhanced with contrast limited adaptive histogram equalization (CLAHE) filter, **c** is the placement of the initial point, **d** is the output after the computation of DP-AC, **e** is the output after transforming the polygon to a binary image and **f** is the final region of interest after superimposing the binary image to the original input image

The proposed DP-AC method required the user to manually assigned the initial point of close contour. In this step, the placement of the initial point is crucial as it will determine the success rate of the image segmentation. Figure 7 shows the sequential steps of the image segmentation process, which involves the image filtration operation, placement of the initial point of closed contour, image conversion from polygon to binary and superimposition of the ROI with the original input image to retain the image pixels.

Based on the proposed segmentation algorithm, two important parameters are assigned before the segmentation process: the number of radial lines and the radius range of the radial line. These two may affect the overall segmentation accuracy. Hence, thirty images were randomly selected to undergo the segmentation process to measure the performance of the proposed algorithm. The average accuracy of the analysis was tabulated in Table 2. The results show that the low number of radius lengths may distort the object's outline, resulting in under-segmentation. In contrast, a higher number may result in over-segmentation as well as longer computation time.

**Table 2:** Segmentation accuracy according to parameters assigned based on P1 and P2

		P1				P2			
Radius length, in pixels	Number of radial lines	Output state	Time (s)	Dice Coefficient (F1 Score)	Jaccard similarity	Output state	Time (s)	Dice Coefficient (F1 score)	Jaccard similarity
80	800	*	1.64	0.85	0.76	*	1.74	0.81	0.73
	1000	*	1.75	0.87	0.78	*	1.83	0.83	0.74
	1200	***	2.36	0.88	0.75	*	1.97	0.85	0.78
100	800	*	2.01	0.85	0.74	*	2.13	0.86	0.75
	1000	**	2.22	0.86	0.77	**	2.29	0.91	0.86
	1200	**	2.42	0.96	0.90	**	2.31	0.94	0.88
120	800	**	2.54	0.94	0.85	*	2.68	0.93	0.84
	1000	*	2.92	0.89	0.73	**	2.77	0.80	0.71
	1200	**	3.26	0.92	0.83	*	3.64	0.92	0.83
140	800	**	2.97	0.92	0.81	*	3.21	0.93	0.84
	1000	**	3.03	0.90	0.79	***	3.98	0.89	0.81
	1200	***	3.93	0.85	0.72	***	4.13	0.84	0.76

*Under-segmented, **well-segmented, ***over-segmented

**Fig. 8:**
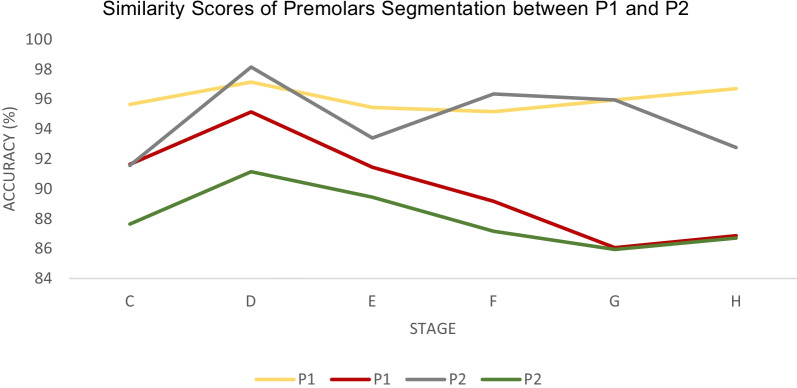
Similarity scores (F1-score and Jaccard index) of the image segmentation output for P1 and P2 according to its developmental stages

A significant correlation is seen between these two variables: the radius length and the number of radial lines assigned. The higher the number of radial lines in any radius length set, the longer the computation time. Besides, less computational time yields most of the output to be under-segmented, while the longer time taken results in over-segmented output. Therefore, the optimal parameters must be chosen based on the ability of the algorithm to segment the ROI in a decent length of time, with the majority of segmentation outputs being well-segmented and having a high segmentation accuracy. Based on the analysis, it can be seen that 100 pixels of radial length associated with 1200 radial lines is superior to other variables assigned. They produce a well-segmented object for almost all of the tested datasets and good segmentation accuracy in a reasonable time. The similarity scores between F1-score and Jaccard index according to the developmental stages was plotted in Fig. 8. On the same figure, it shows that the average segmentation accuracies are above 80%, indicating promising output.

**Fig. 9:**
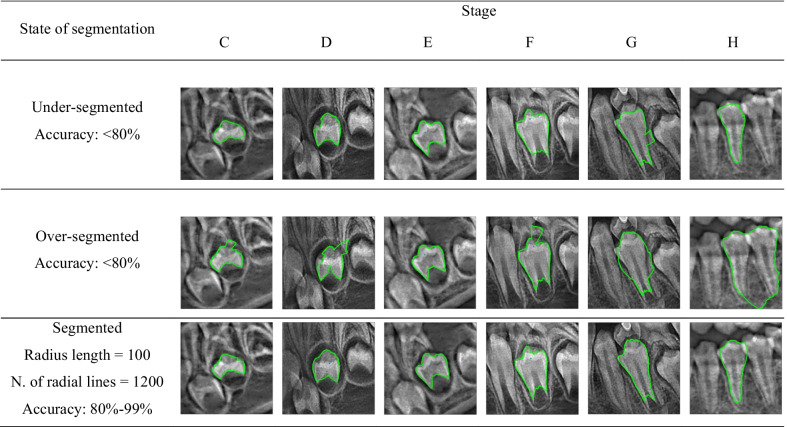
Under-segmented, over-segmented and segmented mandibular first premolar according to Demijian’s staging system

Meanwhile, Fig. 9 shows an example of segmentation results from stages C to H, divided into three groups: under-segmented, over-segmented, and segmented. The following section will discuss the significance of performing the image segmentation before the classification operation and the effect of performing the image segmentation on the classification accuracy.

### Convolutions neural network model selection

The convolutions neural network model was selected based on the lowest validation loss obtained after the model optimization process. The algorithm was run using the Python TensorFlow and Keras library while the output was visualized in the Python Tensorboard. Several parameters need to be assigned in the algorithm before performing the model selection which is as follows; $\mathrm{Dense layers }= [0, 1, 2]$, $\mathrm{layer}\_\mathrm{sizes }= [16, 32, 64]$ and $\mathrm{conv}\_\mathrm{layers }= [1, 2, 3]$.

**Fig. 10:**
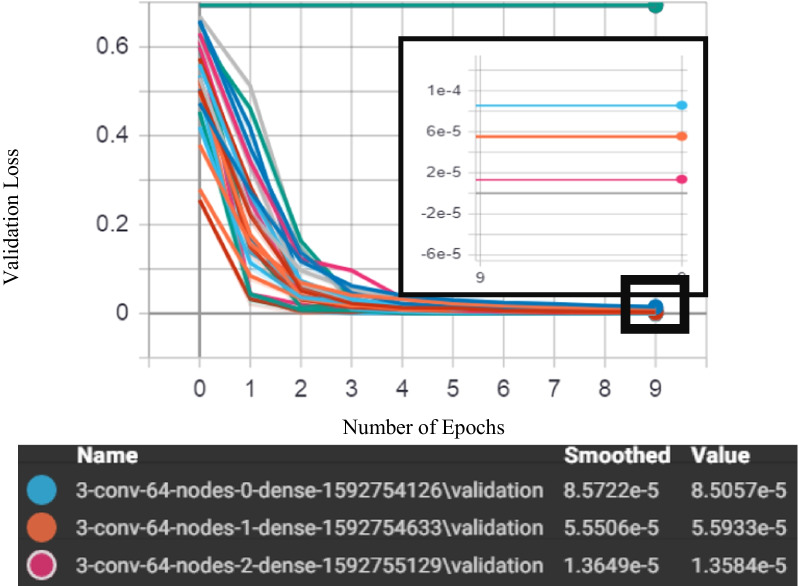
Selection model of covolutional neural network

Figure 10 shows the output for the convolutions neural network model optimization where the best three models were presented in the bounding box with a black colour where the model with 3-convolutional layers-64 nodes-2 dense layers indicates the best convolutions neural network model for our datasets by presenting the lowest validation loss among all tested model followed by the 3-convolutional layers-64 nodes-1 dense layers and 3-convolutional layers-64 nodes-0 dense layers.

### Optimizers selection

**Fig. 11:**
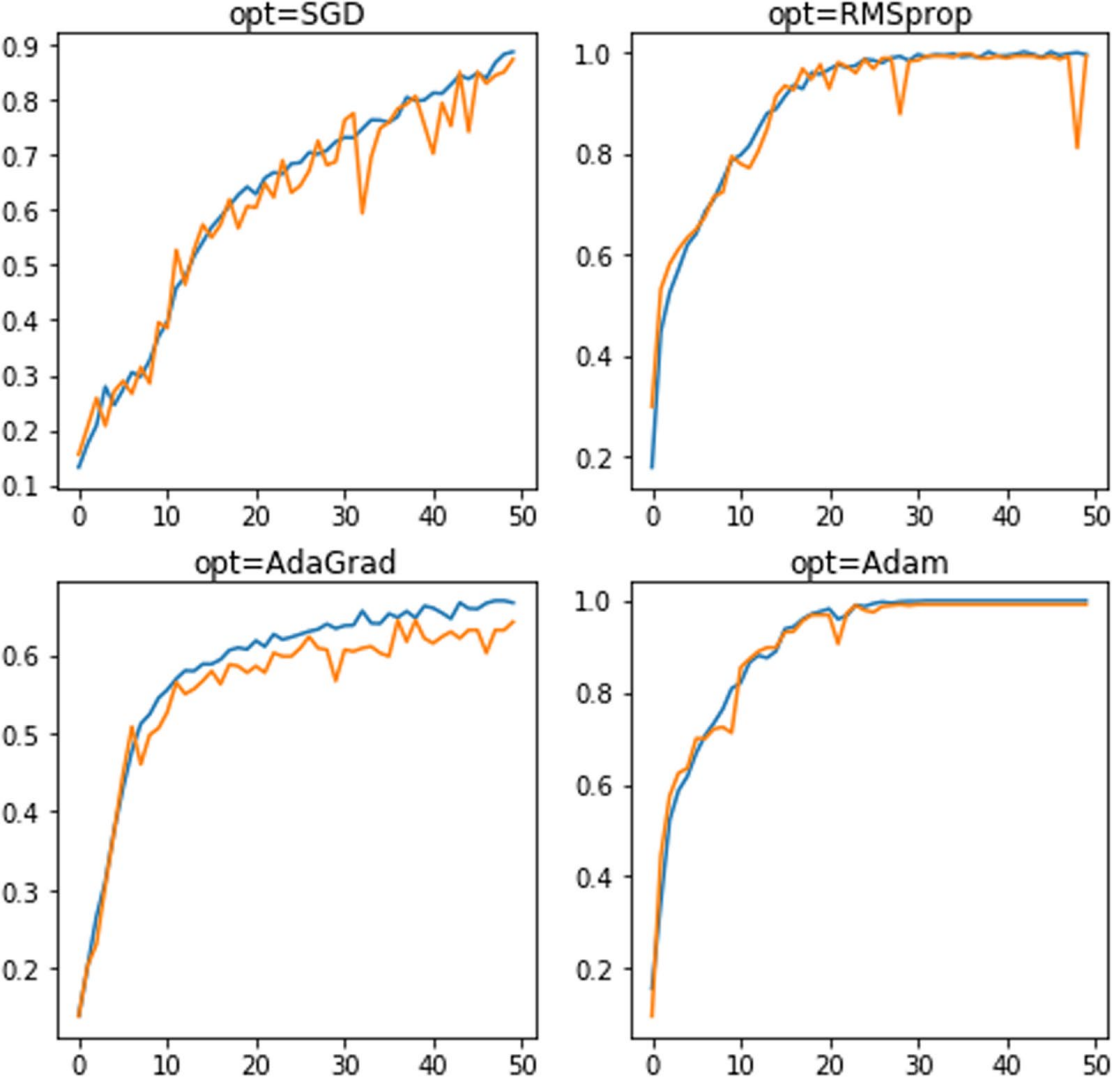
Optimizer selection

In this analysis, “Adam” optimizer was selected with the default hyperparameter values which are ${\beta }_{1}=0.9$, ${\beta }_{2}=0.999$ and learning rate, $\varepsilon ={10}^{-3}$ to reduce the overall loss function proposed by the CNN model. The "Adam" optimizer was chosen by conducting cross-validation on the datasets. Four forms of optimizers were tested, including "Adam," "SGD," "RMSProp" and "AdaGrad," where the CNN model ran across 50 epochs. Figure 11 shows the efficiency of the network, depending on the optimizing assignment. Among all, "Adam" reveals the most stable optimizer as it converged in 30 epochs.

### Accuracies and losses

**Fig. 12:**
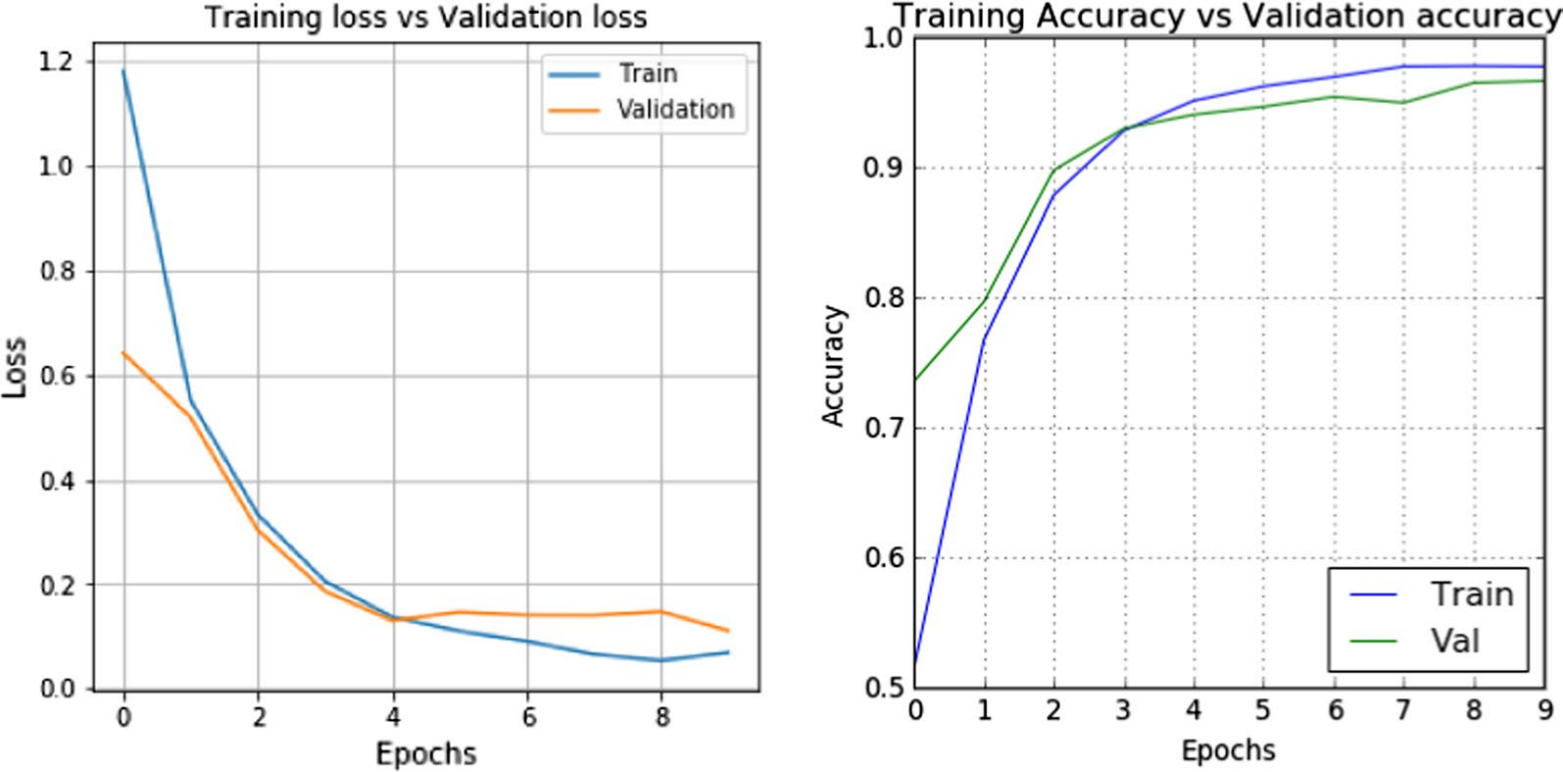
Visualization of losses and accuracies

A learning curve is a plot of model learning performance over time. Figure 12 shows the behaviour of the proposed DCNN model. A plot of learning curves shows slightly overfitting as the plot of validation loss decreases to the point that happened at the 4^th^ epoch and begins increasing again. The proposed model has an accuracy of 97.74% on the training set and 96.63% on the accuracy plot's validation set. In other words, the model is expected to perform classification with 96.63% accuracy on new data. An extensive neural network trained on relatively small datasets will overfit training data. Adding a dropout layer is an easy way to avoid overfitting.

Table 3 demonstrates the learning accuracy of the dropout layer in the proposed DCNN model. Without dropout, the accuracy of the training is slightly higher than the accuracy of the validation while adding 10% (0.1) dropout, both the accuracy of the training and the accuracy of the validation is synchronized. However, the increase in dropout values can reduce the accuracy of learning. Furthermore, image segmentation using the DP-AC algorithm helps enhance the visibility of the image features in the ROI while suppressing the image's background noise. Hence, this may impact the classification accuracy of the test dataset.

**Table 3:** Training and validation accuracy with and without dropout layer

	Training accuracy	Validation accuracy
Without dropout	97.74	96.63
With dropout (*p* = 0.1)	96.17	96.17
With dropout (*p* = 0.5)	86.45	91.42
With dropout (*p* = 0.7)	78.60	90.66
With dropout (*p* = 0.9)	52.45	73.35

**Fig. 13:**
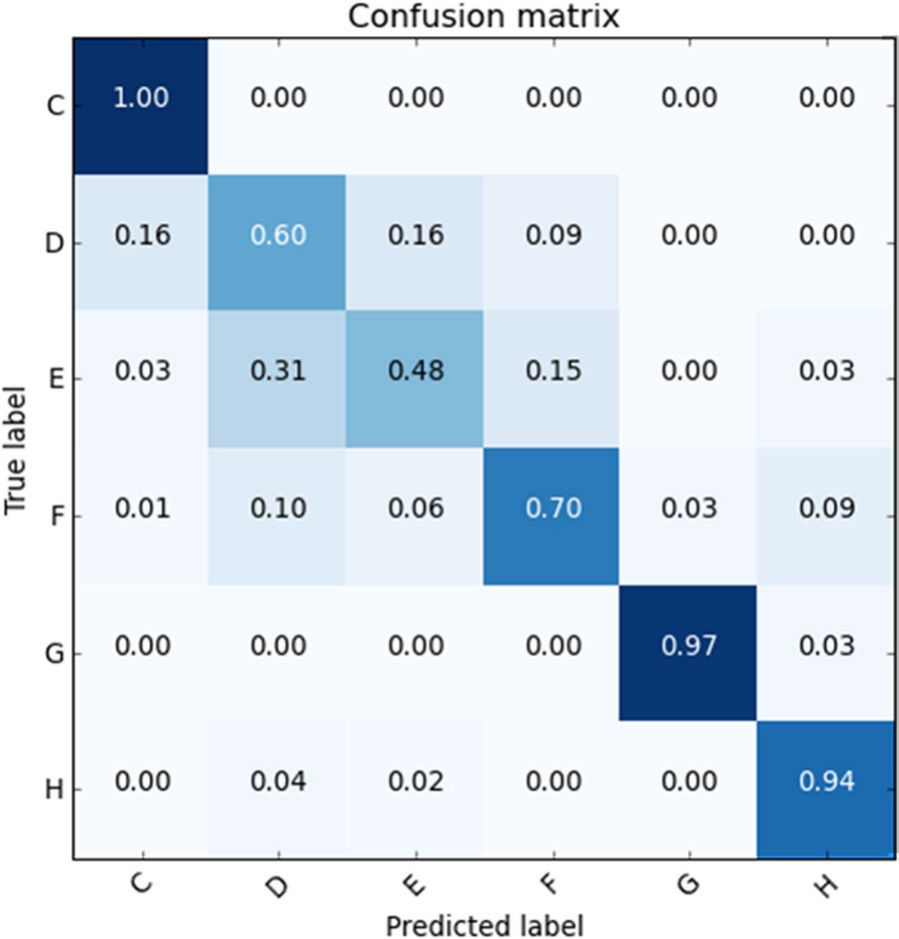
Classification accuracy of the proposed DCNN model

In machine learning, a confusion matrix depicts the summary of prediction results on a classification problem. It is also often used to describe the performance of the classifier, in this case, the proposed DCNN model presented in this study. This study categorized as a multiclass classification problem as the desired output would be to classify images into six categories that involve six stages of dental development. Thus, the confusion matrix would be a 6 × 6 matrix, as shown in Fig. 13. The classification accuracy on a set of test data obtained is 0.781. The confusion matrix reveals no misclassification in stage C, while small errors were detected in stages G and H. However, the accuracy appears to be quite promising. Meanwhile, most misclassified stages minimally happened in the neighbouring stages only for stages D, E, and F.

Cohen's Kappa was also employed in the context of a classification model to compare the machine learning model predictions with the manually established scores. It is also used to evaluate the performance of a classification model. Table 4 shows the allocated stages by the machine (rows) and by the human observers (columns). The value for Kappa is 0.58, indicating a moderate level of agreement.

**Table 4:** Cross-tabulation of the stages assigned using the ground truth data (rows) and the DCNN model (columns)

*Stages*	C	D	E	F	G	H
C	**0.96**	0.04	0	0	0	0
D	0.11	**0.63**	0.13	0.14	0	0
E	0	0.16	**0.56**	0.28	0	0
F	0	0	0.15	**0.66**	0.19	0
G	0	0	0	0.03	**0.94**	0
H	0	0	0	0	0.09	**0.91**

Kappa index: 0.58, Overall accuracy: 0.77

## Discussion

Digital image processing (DIP) is the process of transforming a digital image using a set of algorithms. It includes simple tasks like picture filtration, as well as more complicated tasks like image segmentation, classifications, emotion identification, anomaly detection, and more. Image segmentation is the process of dividing a digital image into many subgroups based on the pixels known as image objects, which can minimize the image's complexity and thus make image analysis easier. It has been utilized in the medical profession for effective and faster diagnosis, as well as the detection of illnesses, tumors, and cell and tissue patterns obtained from various medical imaging techniques such as radiography, MRI, CT scan, ultrasound, and so on.

The proposed method, which requires segmentation of mandibular premolar teeth before image classification, has some flaws that need to be worked out in future studies. The original images for digital panoramic dental imaging were of various sizes and resolutions. The input images should be normalized as a result. When the chosen parameters affect image classification accuracy, using the proper image normalization techniques is crucial. Resizing an image, for example, may increase its general size but reduce its resolution and distort the edges of the ROI, lowering classification accuracy. Furthermore, the dental developmental stages A and B were omitted and will not be tested due to the limited number of datasets. It is because the dental development has proceeded to the stage of the lower-bound chronological age (stages A and B). Hence, the input dataset for image classification only involved the image of mandibular premolars' developmental stage from stage C onwards until stage H.

The quality of digital panoramic dental imaging varies greatly from one patient to another, depending on the patient's position during the treatment as well as the expertise of the human operators. As a result, rather of going through the automated procedure, a semi-automated technique has been implemented. For example, getting a decent quality image requires proper placement of a bite-blocker and the patient's head. Furthermore, the panoramic x-ray can give a somewhat fuzzy image from time to time, making precise measurements of your teeth and jaw problematic. As a result, due to the wide range of data sources, developing a fully automated system for age estimation can be difficult.

Image segmentation is employed in this study to segment the first and second mandibular of permanent teeth before performing image classification, based on the adaptability of digital image processing technique. In this entire semi-automated approach, the use of the DP-AC method has proven to be successful. The easy steps to implement the DP-AC method are presented in Fig. 5, making this approach handleable by the end-user. Rather than constructing a completely automated system, which would require a considerable financial investment, a semi-automated system has produced promising results and satisfactory performance in the assessment of dental age.

The dental age for permanent teeth can be estimated by monitoring dental calcification development using radiographic images. Demirjian et al. suggested the staging of teeth based on the development of the teeth' outline instead of its proportions using the lower-left seven permanent teeth, except the third molar. The implementation of DCNN for the classification of dental stages yields promising results as the accuracy of the classification obtained is very high in some of the predicted stages.

Misclassification has occurred due to the variety of factors that may be linked to the deep neural network's effectiveness and the significance of assigned parameters or other factors related to dental morphology that affect the neural network's ability to achieve a useful classification. The proposed DCNN model was explicitly built based on our datasets. The experiment was performed by assigning parameters to a network model involving layer sizes, several dense layers and convolution layers. As a result, the stage classification accuracy obtained was 0.78. A pre-trained model of CNN which are DenseNet201 and AlexNet was adapted by Merdietio et al. [15], Banar et al. [28] and De Tobel et al. [14], respectively. Based on the performance, the proposed method performed superior compared to the other three methods, which are 0.61, 0.54 and 0.51, respectively.

Moreover, the misclassification of the test datasets is likely due to the behavior of the dataset itself. For example, the new test data implemented did not adequately reflect the broader domain cases. Therefore, this would potentially impact the accuracy of the test. However, based on Table 4, the proposed DCNN model looks promising as more than 90% of the test data in stages C, G and H were correctly allocated. Lower accuracies in stages D, E and F may be due to the significant variation of the morphological structure of dentition between stages. As the human interpretations of allocated stages are highly dependent on skills and experiences, a mutual agreement could not be achieved in some observation samples. Hence, the kappa value obtained is 0.58, indicating moderate agreement. However, most misclassified stages were seen only in the neighboring stages. Although it was not a perfect agreement, the proposed DCNN model showed a robust network. There is no sign of whether the model is over-or underfitting detected during the learning process, whereby the training accuracy was noted higher than the validation and testing accuracy.

Common challenges in deep learning models include the lack of data available for training, model overfitting, model underfitting and high training time. In this research, data augmentation techniques involving image resizing, rescaling, spinning, flipping, cropping, filtering, and brightness modification have been used to increase the number of training datasets. This technique is achieved using the open-source Python preprocessing package known as Scikit-image. Therefore, the model was able to perform well and escape under-fitting in the validation collection.

Model overfitting is the most common problem that data scientist has faced in the field of machine learning [29]. Introduction of the dropout feature to the design of the model is one of the methods used to resolve the overfitting problem. Some of the neurons in the neural network were switched off using the dropout. For example, in an experiment, a drop of 0.1 to a layer initially had 30 neurons that removed three neurons out of its total number of neurons. As a result, a less complicated architecture was obtained, and the model will not learn the intricate pattern. Overall, it can be argued that the DCNN structure plays a critical role in the classification process as it determines the overall performance of the automated stage allocation.

## Conclusion

The application of digital imaging and Keras-based deep learning techniques used by the DP-AC and convolutions neural network algorithms to segment and identify premolars provides promising results for semi-automated forensic dental staging in the future. The techniques used to optimize the DCNN model can be expanded by adding some other hyperparameters to pick a better model with better performance. In addition, wide ranges of datasets will be tested on the proposed model to reduce the inter-rater discrepancy and enhance reproducibility.

## Data Availability

The datasets generated and analyzed during the current study are not publicly available due to the security of data but are available from the corresponding author on reasonable request.

## Abbreviations

DCNN: Deep learning convolutional neural networks
DP-AC: Dynamic programming of active contour
P1: Left mandibular first permanent premolars
P2: Left mandibular second permanent premolars
DP-GICOV: Dynamic programming of gradient inverse coefficient of variation
CV: Chan-Vese
HTGkFCM: Hyperbolic tangent gaussian kernel fuzzy C-means clustering
